# Methotrexate therapy associates with reduced prevalence of the metabolic syndrome in rheumatoid arthritis patients over the age of 60- more than just an anti-inflammatory effect? A cross sectional study

**DOI:** 10.1186/ar2765

**Published:** 2009-07-16

**Authors:** Tracey E Toms, Vasileios F Panoulas, Holly John, Karen MJ Douglas, George D Kitas

**Affiliations:** 1 Department of Rheumatology, Dudley Group of Hospitals NHS Trust, Russells Hall Hospital, Dudley, West Midlands, DY1 2HQ, UK; 2 ARC Epidemiology Unit, Manchester University, Oxford Road, Manchester, M13 9PT, UK

## Abstract

**Introduction:**

The metabolic syndrome (MetS) may contribute to the excess cardiovascular burden observed in rheumatoid arthritis (RA). The prevalence and associations of the MetS in RA remain uncertain: systemic inflammation and anti-rheumatic therapy may contribute. Methotrexate (MTX) use has recently been linked to a reduced presence of MetS, via an assumed generic anti-inflammatory mechanism. We aimed to: assess the prevalence of the MetS in RA; identify factors that associate with its presence; and assess their interaction with the potential influence of MTX.

**Methods:**

MetS prevalence was assessed cross-sectionally in 400 RA patients, using five MetS definitions (National Cholesterol Education Programme 2004 and 2001, International Diabetes Federation, World Health Organisation and European Group for Study of Insulin Resistance). Logistic regression was used to identify independent predictors of the MetS. Further analysis established the nature of the association between MTX and the MetS.

**Results:**

MetS prevalence rates varied from 12.1% to 45.3% in RA according to the definition used. Older age and higher HAQ scores associated with the presence of the MetS. MTX use, but not other disease modifying anti-rheumatic drugs (DMARDs) or glucocorticoids, associated with significantly reduced chance of having the MetS in RA (OR = 0.517, CI 0.33–0.81, *P *= 0.004).

**Conclusions:**

The prevalence of the MetS in RA varies according to the definition used. MTX therapy, unlike other DMARDs or glucocorticoids, independently associates with a reduced propensity to MetS, suggesting a drug-specific mechanism, and makes MTX a good first-line DMARD in RA patients at high risk of developing the MetS, particularly those aged over 60 years.

## Introduction

Rheumatoid arthritis (RA) patients have a reduced life expectancy and higher mortality rates than the general population [1,2], with cardiovascular disease (CVD) accounting for approximately half of this [3,4]. Although traditional cardiovascular risk factors such as hypertension [5,6], central obesity [7,8] and insulin resistance [9] may occur more frequently among RA patients, this does not fully account for the rates of CVD observed [10], and besides genetic predisposition [11], novel risk factors and mechanisms, including systemic inflammation *per se*, have also been implicated [12].

The metabolic syndrome (MetS) reflects a clustering of classical cardiovascular risk factors including insulin resistance, central obesity, elevated blood pressure, high triglyceride (TG) levels and low levels of high-density lipoprotein (HDL) [13]. The MetS has been identified as an independent cardiovascular risk factor, conferring risk above and beyond the sum of its individual components [14], although this has recently been questioned [15]. The MetS has been shown to be highly prevalent among American patients with RA, with rates being four times those reported in the general population [16]. In contrast, another study among Mediterranean RA patients also showed a high MetS prevalence but failed to demonstrate a significant difference from local general population controls [17].

To date, five definitions for the MetS have been developed: The National Cholesterol Education Programme (NCEP) 2004 [18] and NCEP 2001 [19], the World Health Organization (WHO) [20], the International Diabetes Federation (IDF) [21] and the European Group for Study of Insulin Resistance (EGIR) [22]. These share many similarities; however, they differ in some of the components, as well as their specified cut-offs and weighting. In the general population, prevalence rates of the MetS have been shown to vary dramatically according to the definition used [23,24], with the IDF classification [21] tending to report the highest and the EGIR classification [22] the lowest within a European study population [25-27]. To date, two comparative studies have been performed in an RA population, both of which found a similar prevalence of the MetS according to the WHO and NCEP 2001 criteria [16,28].

Several of the individual components of the MetS have been shown to be influenced by demographic, anthropometric and RA-specific factors [6,8,29], but there has been very little work aimed at identifying factors that may be associated with the presence of MetS as a whole in patients with RA [30]. Such associations may be key to tackling MetS and reducing CVD-related morbidity and mortality in RA. Studies have demonstrated significant reductions in CVD-related mortality in patients treated with methotrexate [31,32]. This finding has been attributed to the potent anti-inflammatory properties of methotrexate. Interestingly, another study in 107 exclusively female RA patients has recently reported a negative association between methotrexate use and the MetS [30]. This relationship was again assumed to be the result of the anti-inflammatory properties exhibited by methotrexate, but no further sub-analyses were performed to confirm or refute this.

In this study we aimed to: (1) assess the prevalence of the MetS in a large RA population according to all definitions currently used, in order to develop a bench-mark allowing comparisons between other relevant studies in the future; (2) to identify demographic, anthropometric and RA-disease specific factors that may be associated with the presence of the MetS in RA patients; (3) to establish if anti-rheumatic drug use (in particular methotrexate), is associated with the presence of the MetS, and whether this occurs in a drug-specific manner or as a result of an overall anti-inflammatory effect.

## Materials and methods

Four hundred RA patients fulfilling the 1987 revised American College of Rheumatology classification criteria [33], were recruited from outpatient clinics at the Dudley Group of Hospitals NHS Foundation Trust between 2004 and 2006 (the Dudley Rheumatoid Arthritis Comorbidity Cohort, the characteristics of which have been previously described in detail) [6,34]. Of them, 387 with a complete dataset required for this study were analysed, and results presented refer to those patients. The study was granted full ethical approval from the local ethics committee and all patients gave their informed written consent prior to commencement of the study.

Patient data was obtained via case note analysis and a face-to-face interview performed by a rheumatologist. The dual approach facilitated the documentation of a detailed history to include: disease course/characteristics (including disease duration), drug use (all anti-rheumatic drugs, glucocorticoid use, cardiovascular drugs and analgesics among others), co-morbid conditions, and family history of rheumatic and cardiovascular diseases. Details of current medication prescriptions were recorded at baseline (no prospective data was collected), and previous anti-rheumatic drug use were recorded via retrospective case note analysis and patient interview. Baseline demographics were recorded and anthropometric characteristics were measured as previously described [9]. Current disease activity and physical function were assessed using the 28-joint disease activity score (DAS28) [35] and the health assessment questionnaire (HAQ) [36], respectively.

Baseline blood samples were obtained from each patient and were analysed in a single laboratory. Blood tests included: C-reactive protein (CRP), erythrocyte sedimentation rate (ESR), fasting lipid profile (total cholesterol (TC), HDL, low density lipoproteins (LDL), TG), rheumatoid factor, anti-cyclic citrullinated peptide antibodies, thyroid function tests), liver function tests, renal function, insulin and fasting glucose. All lipid components were analysed using the Vitros ^® ^5,1FS chemistry system (Ortho Clinical Diagnostics, Markham, Ontario, Canada), with multilayered slides used to measure TC, HDL, and TGs, whereas a dual chamber package was used to assess LDL, apolipoprotein (Apo) A and ApoB. Insulin resistance was evaluated from fasting glucose and insulin using the Homeostasis Model Assessment of Insulin Resistance (HOMA IR) [37] and the Quantitative Insulin Sensitivity Check Index (QUICKI) [38], and was defined as the presence of diabetes mellitus or HOMA IR of 2.5 or more or QUICKI of 0.333 or less. Renal function assessment was made by estimation of glomerular filtration rate according to the Modification of Diet in Renal Disease equation [39].

For the purposes of this study, the prevalence of the MetS was analysed according to all existing definitions (NCEP 2004, NCEP 2001, WHO, IDF, EGIR; Table 1) in order to establish the range of discrepancy between them. For further analysis of the predictors of the metabolic syndrome only the NCEP 2004 definition is presented, as this is most up to date and widely used definition reported in the literature, thus allowing comparisons to be drawn with other studies.

**Table 1:** A summary of the definitions of the metabolic syndrome

	**NCEP 2004 **[18]	**NCEP 2001 **[19]	**WHO **[20]	**EGIR **[22]	**IDF **[21]
**Number of criteria**	Three or more of:	Three or more of:	And two or more of:	And two or more of:	And two or more of:
**Obesity**	WC ≥ 102 cm (men), WC ≥ 88 cm (women)	WC ≥ 102 cm (men), WC ≥ 88 cm (women)	BMI > 30 and/or WHR > 0.9 (men), WHR > 0.85 (women)	WC ≥ 94 cm (men, WC ≥ 80 cm (women)	WC ≥ 94 cm men, WC ≥ 80 cm women*
**Hypertension (mmHg)**	≥ 130/85**	≥ 130/85**	≥ 140/90	≥ 140/90**	≥ 130/85**
**Dyslipidaemia:** **HDL-C (mmol/L)**	< 1.0 (men), < 1.3 (women)**	< 1.0 (men), < 1.3 (women)**	< 0.9 (men) < 1.0 (women) or	< 1.0**	< 1.0 (men) < 1.3 (women)**
**TG (mmol/L)**	≥ 1.7**	≥ 1.7**	≥ 1.7	> 2.0**	> 1.7**
**Glucose intolerance or fasting plasma glucose (mmol/L)**	≥ 5.6**	≥ 6.1**	≥ 6.1, ***DM, IGT, IR***	≥ 6.1 (excludes diabetics) ***Insulin in top 25%***	≥ 5.6**
**Albumin/creatinine ratio (mg/L)**	N/A	N/A	≥ 30	N/A	N/A

***Text in bold italics***: prerequisite for diagnosis, in addition to the number of other criteria needed to be met. ** or treated for abnormality, * cut-off values differ according to ethnic origin.

BMI = body mass index; DM = diabetes mellitus; EGIR = European Group against Insulin Resistance; HDL-C = high-density lipoprotein-cholesterol; IDF = International Diabetes Federation; IGT = impaired glucose tolerance; IR = insulin resistance; N/A = not applicable;NCEP = National Cholesterol Education Programme; TG = triglyceride; WC = waist circumference; WHO = World Health Organization; WHR = waist hip ratio.

### Statistical analysis

This was carried out using SPSS 15.0 (SPSS Inc, Chicago, IL, USA). The distribution of each variable was examined using Kolmogorov-Smirnov function. Results are expressed as mean ± standard deviation, median (25^th ^to 75^th ^percentile), or percentages, as appropriate. For the univariate analysis, chi-squared, t-test and Mann-Whitney U tests were used to test categorical, normally and not normally distributed data, respectively. The independence of the predictors of the MetS was tested in the multivariate models using binary logistic regression.

## Results

### Descriptive characteristics of study population

The study population comprised of 72.9% females (282/387) and had a median age of 63.1 years. Patients had a median disease duration of 10 years, and had moderate disease activity (mean DAS28 score 4.2).

Disease-modifying anti-rheumatic drugs (DMARDs) were widely prescribed among this cohort (340/387), either as monotherapy (218/387) or combination therapy (122/387). The breakdown of DMARD usage was: 218 (56%) patients were taking methotrexate, 114 (29.5%) sulphasalazine, 77 (19.9%) hydroxychloroquine and 16 (4.1%) leflunomide. Biologic therapy and glucocorticoids were prescribed in 45 (11.6%) and 56 (14.5%) patients, respectively. The use of other drugs known to influence components of the MetS included: statins in 83 (21.4%), anti-hypertensives in 171 (44.2%) and NSAIDs/cyclo-oxygenase-II inhibitors in 108 (27.9%) patients.

### Prevalence of the metabolic syndrome according to definition used

There was great diversity in the reported prevalence rates according to the definition used (Table 2). The prevalence ranged from 12.1% to 45.3%, with EGIR reporting the lowest rate, the IDF criteria reporting the highest rate, and the currently most commonly used NCEP 2004 criteria reporting a rate of 40.1%. A small variation in the total number of patients included for analysis of prevalence of the metabolic syndrome according to each definition was observed. This phenomenon was the result of incomplete data on a few patients. The prevalence of the MetS increased with age up until the seventh decade and fell off thereafter, and was similar in males and females (*P *= 0.429; Table 3).

**Table 2:** Prevalence of metabolic syndrome according to definition used

**Definition of MetS used**	**Prevalence**			
	**Total**	**Males**	**Females**	***P *value**

IDF n (%)	159 (45.3)	49 (52.7)	110 (42.6)	0.095
NCEP 2004 n (%)	156 (40.1)	45 (42.5)	111 (39.2)	0.563
NCEP 2001 n (%)	149 (38.3)	42 (40.0)	107 (37.7)	0.676
WHO n (%)	70 (19.4)	25 (25.5)	45 (17.2)	0.075
EGIR n (%)	47 (12.1)	24 (22.6)	23 (8.2)	< 0.001

EGIR = European Group for Insulin Resistance; IDF = International Diabetes Federation; MetS = metabolic syndrome; NCEP = National Cholesterol Education Programme; WHO = World Health Organization.

**Table 3:** Prevalence of the metabolic syndrome in specific age ranges

	**Prevalence**				
	**NCEP 2004** **n = 156**	**NCEP 2001** **n = 149**	**WHO** **n = 70**	**IDF** **n = 159**	**EGIR** **n = 47**

Age < 40 years n (%)	2 (0.5)	2 (0.5)	2 (0.6)	3 (0.9)	0 (0)
Age 40 to 49 years n (%)	12 (3.1)	12 (3.1)	4 (1.1)	12 (3.4)	0 (0)
Age 50 to 59 years n (%)	32 (8.2)	29 (7.5)	15 (4.2)	32 (9.1)	12 (3.1)
Age ≥ 60 years n (%)	110 (28.3)	106 (27.2)	49 (13.6)	112 (31.9)	35 (9.0)

EGIR = European Group for Insulin Resistance; IDF = International Diabetes Federation; NCEP = National Cholesterol Education Programme; WHO = World Health Organization.

### Associations of the metabolic syndrome in patients with RA

Results presented are only for the MetS as defined by NCEP 2004, but were very similar using any of the other definitions, despite the difference in prevalence.

In univariate analysis, patients with the MetS were significantly older (*P *= 0.001), had shorter disease duration (*P *= 0.008), higher ESR (*P *= 0.006), higher HAQ scores (*P *= 0.036) and significantly less of them were treated with methotrexate (*P *= 0.001), compared with those who did not have the MetS (Table 4).

**Table 4:** Demographic, clinical and laboratory characteristics of the study population (NCEP 2004 definition used)

	**Total (n = 387)**	**MetS present (n = 156)**	**MetS abscent** **(n = 232)**	***P *value**
***General demographics***				
Age (years)	63.1 (55.5 to 69.6)	65.3 (58.2 to 69.8)	61.4 (51.9 to 75.4)	0.001
Sex female n (%)	282 (72.9)	110 (71)	172 (74.1)	0.429
***RA characteristics***				
***General characteristics***				
RF positive n (%)	287 (75.9)	121 (79.1)	166 (73.8)	0.236
Anti-CCP positive n (%)	250 (67.6)	101 (68.2)	149 (67.1)	0.821
Disease duration (years)	10 (4 to 18)	9 (4 to 18.5)	10 (4 to 17)	0.008
***Disease activity***				
CRP (mg/L)	8 (5 to 20)	9 (5 to 20)	8 (4 to 18)	0.155
ESR	21 (9 to 37)	23 (15 to 40)	18 (8 to 32)	0.006
DAS28	4.2 +/- 1.4	4.25 +/- 1.29	4.14 +/- 1.44	0.437
***Disease severity***				
HAQ	1.5 (0.63 to 2.13)	1.63 (0.88 to 2.25)	1.5 (0.38 to 2)	0.036
EAD n (%)	257 (66.4)	11 (71.6)	146 (62.9)	0.076
Joint replacement surgery n (%)	276 (71.3)	110 (71)	166 (71.6)	0.901
***Medication***				
Methotrexate n (%)	218 (56.0)	72 (46.5)	148 (63.8)	0.001
Sulphasalazine n (%)	114 (29.5)	46 (29.7)	68 (29.3)	0.938
Hydroxychloroquine n (%)	77 (19.9)	26 (16.8)	51 (22)	0.209
Anti-TNF n (%)	45 (11.6)	20 (12.9)	25 (10.8)	0.522
Leflunomide n (%)	16 (4.1)	8 (5.2)	8 (3.4)	0.407
Prednisol medium dose n (%)	56 (14.5)	28 (12.1)	28 (18.1)	0.241
NSAIDs/COX-II n (%)	108 (27.9)	37 (23.9)	71 (30.6)	0.148
Anti-hypertensives n (%)	171 (44.2)	107 (69)	64 (24.6)	< 0.001
Statin/fibrate n (%)	83 (21.4)	80 (51.6)	3 (1.3)	< 0.001
***Risk factors for the MetS***				
Waist (cm)	97.7 +/- 13.13	103.5 +/- 13.13	93.8 +/- 11.69	< 0.001
Triglycerides (mmol/L)	1.2 (1 to 1.6)	1.5 (1.1 to 2.1)	1.1 (0.9 to 1.4)	< 0.001
Systolic BP (mmHg)	140 (127 to 154.5)	144 (132.5 to 159.5)	65.3 (58.1 to 69.8)	< 0.001
Diastolic BP (mmHg)	78.9 +/- 11.18	79.68 +/- 11.14	78.56 +/- 11.05	0.331
HDL (mmol/L)	1.6 (1.3 to 1.8)	1.4 (1.1 to 1.7)	1.6 (1.4 to 1.9)	< 0.001
Insulin resistance n (%)	140 (37.2)	87 (62.1)	53 (37.9)	< 0.001
**Criteria for MetS**				
WaistM n(%)	230 (65.7)	122 (86.5)	108 (51.7)	< 0.001
TriglyceridesM n(%)	147 (38)	125 (80.6)	22 (9.5)	< 0.001
HypertensionM n(%)	311 (80.4)	151 (97.4)	160 (69)	< 0.001
HDLM n(%)	136 (35.1)	116 (74.8)	20 (8.6)	< 0.001
FPGM1 n(%)	57 (14.8)	47 (30.5)	10 (4.3)	< 0.001
Albumin/Creatinine ratio	42 (10.9)	21 (10.6)	21 (14.6)	0.269

Results expressed as percentages, median (25^th ^to 75th percentile values) or mean ± standard deviation as appropriate. insulin resistance = homeostasis model assessment ≥ 2.5 or quantitative insulin sensitivity check index = 0.333; waist M = waist circumference > 102 cm in males and > 88 cm in females; triglyceridesM = triglycerides ≥ 1.7 mmol/L or on drug treatment for elevated triglycerides, hypertensionM = systolic BP ≥ 130/85 mmHg or on antihypertensive medication; HDLM = high-density lipoprotein level < 1.0 mmol/L in males or < 1.3 mmol/L in females; FPGM = fasting plasma glucose ≥ 6.1 or on drug treatment for elevated blood glucose.

anti-CCP = anti-cyclic citrullinated peptide; BP = blood pressure; COX-II = cyclooxygenase II inhibitors; CRP = C-reactive protein; DAS = Disease Activity Score; EAD = extra-articular disease; ESR = erythrocyte sedimentation rate; HAQ = Health Assessment Questionnaire; HDL = high-density lipoprotein; MetS = metabolic syndrome; NCEP = National Cholesterol Education Programme; NSAIDs = non-steroidal anti-inflammatory drugs; RA = rheumatoid arthritis; RF = rheumatoid factor; TG = triglycerides; TNF = tumour necrosis factor.

The independence of each of these associations (and for completeness also sex) were tested in a multivariate logistic regression model. Older age (β = 0.034, *P *≤ 0.001), higher HAQ scores (β = 0.335, *P *= 0.024) and less methotrexate use (β = -0.663, *P *= 0.001) values remained significant independent predictors of the presence of the MetS in RA patients. Patients on methotrexate had half the odds of having the metabolic syndrome compared with those not taking methotrexate (odds ratio (OR) = 0.525, 95% confidence interval (CI) = 0.96 to 1.56, *P *= 0.003). The odds were not significantly altered when other DMARD, anti-TNF therapies, glucocorticoid use and NSAID medications were added to the model (OR = 0.517, 95% CI = 0.33 to 0.81, *P *= 0.004; Table 5).

**Table 5:** Odds ratios for having the metabolic syndrome in patients receiving methotrexate compared with those not on methotrexate

	**Odds ratio**	**95% confidence interval**	***P *value**
Crude	0.483	0.32 to 0.73	0.001
Model a	0.505	0.33 to 0.77	0.001
Model b	0.525	0.34 to 0.80	0.003
Model c	0.517	0.33 to 0.80	0.004

Crude = unadjusted model

Model a = adjusted for age and sex

Model b = adjusted for age, sex, disease duration, erythrocyte sedimentation rate and health assessment questionnaire score

Model c = adjusted for age, sex, disease duration, erythrocyte sedimentation rate, health assessment questionnaire score, sulphasalazine, hydroxyxhloroquine, leflunomide, anti-tumour necrosis factor therapy, glucocorticoid use and NSAID use.

The multivariate model was repeated, replacing ESR with DAS28 score and subsequently with CRP, to check for any differences among these potential confounders. The results were not found to differ significantly by using DAS28 or CRP instead of ESR (OR = 0.480, 95% CI = 0.26 to 0.88, *P *= 0.017; or OR = 0.466, 95% CI = 0.26 to 0.83, *P *= 0.010, respectively).

### Methotrexate and the metabolic syndrome

**Figure 1:**
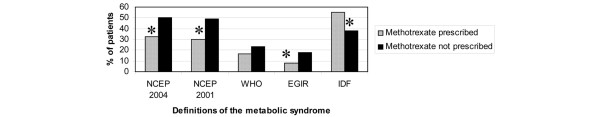
**The relationship between methotrexate use and the presence of the metabolic syndrome according to the definition used**. * *P *< 0.05. EGIR = European Group Against Insulin Resistance; IDF = International Diabetes federation; NCEP = National Cholesterol Education Programme; WHO = World Health Organisation.

**Figure 2:**
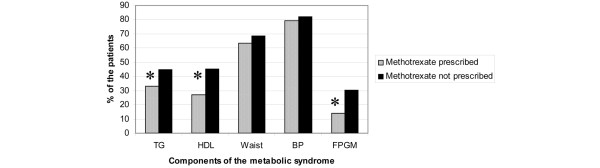
**Frequency of individual components of the metabolic syndrome (NCEP 2004) among patients taking methotrexate and not taking methotrexate**. * *P *< 0.05. BP = blood pressure; FPGM = fasting plasma glucose > 5.6 mmol/L; HDL = high density lipoproteins; NCEP = National Cholesterol Education programme; TG = triglycerides.

Methotrexate was found to be an independent predictor for the MetS according to all definitions except WHO (Figure 1). Methotrexate use was associated with improvements in lipid parameters and fasting plasma glucose levels, with lower TG levels (*P *= 0.019), higher HDL levels (*P *≤ 0.001), and lower fasting plasma glucose levels (*P *≤ 0.001; Figure 2). Methotrexate use did not associate with waist circumference, blood pressure or insulin resistance. No significant association was found between previous methotrexate use and having the MetS.

## Discussion

In this study we confirm that the MetS is highly prevalent in RA (in up to 45.3% of patients) but its prevalence depends on the definition used, in a very similar manner to that seen in the general population, with the IDF criteria reporting the highest and the EGIR criteria the lowest rates. We also demonstrate for the first time that, irrespective of the definition used, factors including older age and disease severity (HAQ) are associated with the presence of the MetS in patients with RA. More importantly, methotrexate therapy appears to significantly decrease the odds of having the MetS, independently of any of these factors, suggesting the possibility of a drug-specific protective mechanism.

To date, four other studies have commented on the prevalence of the MetS in patients with RA, reporting prevalence rates ranging from 14% to 44% [16,17,28,30]. Such diversity can be explained by differences in the baseline characteristics and disease characteristsics. Overall, we have reported similar prevalence rates according to the NCEP 2001 as other investigators (38.3%). However, we report a lower prevalence when using the WHO criteria (19.4%) and find this discrepancy difficult to explain, particularly in the context of the relative concordance between the prevalence rates defined by the NCEP and WHO criteria in two other studies [16,28].

In this study we observed similar prevalence rates of the MetS among males and females (*P *= 0.429). This was consistent across all definitions of the metabolic syndrome, apart from the EGIR classification, which diagnosed significantly more males than females (*P *< 0.001). These findings differ from those observed in the general population, where age-matched females have been reported to have significantly higher rates of the MetS [40]. This discrepancy may be a consequence of the ongoing inflammatory burden in the RA population, altering some of the components of the metabolic syndrome.

The factors found to associate independently with the metabolic syndrome in RA included older age, higher HAQ scores and less methotrexate usage. The association with older age is not surprising, because in the general population the MetS has been shown to affect primarily older subjects, as a consequence of age-related modification of some of its components [41]. Higher HAQ scores are also likely to associate with the MetS in RA, because patients with more severe disabling disease are likely to lead a less active lifestyle, resulting in increased obesity and alterations in the lipid profile [42,43].

One of the most interesting findings from this study, however, is the negative association between methotrexate use and the presence of the MetS, which suggests that MTX may protect against its development. This association has also recently been observed in a study by Zonana-Nacach and colleagues [30]. In that study, this association was assumed to be purely due to the anti-inflammatory effect of MTX, leading to modification of the components that collectively make up the MetS, although no data were presented to support this contention. The results of our study suggest that any protective effect of methotrexate is likely to be drug-specific, and not the result of a generic anti-inflammatory effect, because it was not observed with any of the other DMARDs. Alongside this, we have recently presented data demonstrating that the use of glucocorticoids is not associated with the presence of the MetS [44], thus again arguing against a potential anti-inflammatory mechanism of action of methotrexate.

In view of patient age acting as an independent predictor for the MetS, we performed a further subanalysis to examine the potential effects of methotrexate on the MetS according to age (age ≥ 60 years versus age < 60 years). This demonstrated that the 'protective' effects of methotrexate on the presence of the MetS are only present in patients over the age of 60 years. These findings are unsurprising given that patients over the age of 60 years have a higher prevalence of the metabolic syndrome, upon which methotrexate can act.

In order to gain further understanding of potential mechanisms that may underlie this phenomenon, we analysed the impact of methotrexate on the individual components of the MetS. Methotrexate use is associated with lower TG, higher HDL and lower fasting glucose levels, but did not appear to be associated with either blood pressure or obesity (as assessed by waist circumference). Although the inflammatory process has been shown to directly modify many of these parameters [29,45,46], this would not explain the specificity of the effect to MTX but not other DMARDs, including biologics, as well as glucocorticoids.

These observations provide interesting insights into the potential mode of action of MTX. One possible mode of action may be through alterations in adenosine concentrations. Extracellular adenosine levels are increased by methotrexate and are known to mediate its anti-inflammatory effect [47,48]. To accompany this, there is evidence that adenosine enhances the effects of insulin on glucose transport and metabolism, and may also alter aspects of lipid metabolism [49]. A recent study has also provided evidence that MTX may offer an atheroprotective effect, through activation of the adenosine A_2A_, thus promoting reverse cholesterol transport [50]. Another possible mode of action may be indirect, in that it may occur not as a consequence of methotrexate use *per se*, but as a consequence of concurrent folic acid supplementation. Folic acid has been shown to suppress plasma homocysteine levels [51]. This may be particularly important in the context of the MetS, which is known to be associated with high homocysteine levels [52]. Insulin resistance is thought to be the key to the underlying pathophysiology of the MetS, and can improve with folate replacement therapy [53]. Thus, suppression of a potential precipitant (homocysteine) via the use of folic acid may protect against the development of the MetS in patients such as these, who take MTX with concurrent folate supplementation. With this in mind, we scrutinised our data further to look at folate and homocysteine levels according to the prescription of methotrexate and the effect these factors have on the development of the MetS. Although significantly higher levels of folate were seen in the patients receiving methotrexate (*P *< 0.001), this did not result in significantly lower levels of homocysteine (*P *= 0.406). We also failed to demonstrate any significant impact of folate levels on the development of the MetS in a binary logistic model (unadjusted OR = 1.006, 95% CI = 0.996 to 1.02, *P *= 0.247, adjusted for age, sex, HAQ OR = 1.005, 95% CI = 0.996 to 1.02, *P *= 0.281). Thus, although this mechanism is still plausible it is not supported by the findings of this study. All of the possible underlying mechanisms of action require further investigation in studies designed specifically for the purpose. However, it remains that the observation described in this study may be important in the clinical context. Methotrexate may be the most appropriate first-line DMARD therapy for RA patients at particular risk of developing the metabolic syndrome, such as the elderly and obese with severe, active RA of relatively short duration.

The association between methotrexate use and the MetS carries further complexities. A strongly significant negative association is apparent with all but the WHO definition. This phenomenon may be explained by differences in the components and cut-off values used in the definitions. The WHO is the only definition to include albumin/creatinine ratio as a criterion, a factor that was not found to be influenced by methotrexate use. Conversely, it could be explained in differences in the sensitivity and specificity of each definition. The use of the NCEP criteria in RA has been questioned over recent years, because it has been found to confer a low sensitivity for predicting insulin resistance [54] and may be less strongly linked to the development of atherosclerosis in RA [28]. Further longitudinal studies are required to confirm or refute these initial findings.

Over recent years, scepticism has arisen over whether the MetS is independently associated with CVD [15]. This issue can only be fully resolved through large-scale prospective trials; however, we attempted to study the association of the MetS and traditional CVD risk factors with CVD. Following adjustment for multiple potential confounders in the present cohort, we found that patients with the MetS had a four-fold increased risk of having CVD compared with those without CVD (OR = 4.069, 95% CI = 2.34 to 7.07, *P *< 0.001). However, apart from diabetes mellitus (OR = 2.76, 95% CI = 1.12 to 6.83, *P *= 0.028), all other components of the MetS had a non-significant association (data not shown).

In addition to the originality of most of the findings, this study has several other strengths. These include the use of all of the existing MetS criteria for the first time in RA, in the largest RA population studied thus far: these data can be used for benchmarking purposes to compare past or future studies, irrespective of the MetS criteria they use. Also, the detailed, prospective data collection minimised selection and recall bias as well as missing data and allowed meaningful sub-analysis with corrections for multiple potential confounders. Despite this, the cross-sectional design is a major limitation and precludes the ability to prove the causality or directionality of the associations found. Our study was also limited to secondary care RA patients from a single geographical location in the UK and did not assess the MetS in local general population controls, although another study of patients with diabetes from the geographically neighbouring (6 miles) area of Wolverhampton suggest that the local population is demographically representative of the total UK population [55]. We cannot therefore claim either that the prevalence of the MetS in patients with RA is higher than in the general population, or that the results regarding prevalence of MetS are generalisable to other populations. However, the associations found with disease characteristics and medication, are unlikely to be subject to geographical differences and the impact they may have on demographics. Although, disease activity was not found to be an independent predictor for the metabolic syndrome, we felt this potentially important association warranted further interrogation, by comparing patients in remission to those with active disease. Unfortunately, the sub-analysis had insufficient power to produce meaningful results. With this in mind we would encourage further longitudinal studies to confirm the drug-specific protective effect of methotrexate against the development of the MetS in other geographical populations, and also in subgroups of RA patients according to their disease activity.

## Conclusions

The MetS is common among RA patients, and may contribute significantly to their excess cardiovascular morbidity and mortality. In order to aggressively address this issue and minimise the associated risk we suggest that the NCEP 2004 criteria should be used as an annual screening tool in RA patients over the age of 60 years to identify RA patients with the MetS. Consideration should be given to using methotrexate with folate supplementation as first-line DMARD therapy in RA patients deemed to be at the highest risk, such as the elderly with early severe active disease.

## Abbreviations

Apo: apolipoprotein; CI: confidence interval; CRP: C-reactive protein; CVD: cardiovascular disease; DAS28: 28-joint disease assessment score; DMARD: disease-modifying anti-rheumatic drugs; EGIR: European Group for Study of Insulin Resistance; ESR: erythrocyte sedimentation rate; HAQ: health assessment questionnaire; HDL: high-density lipoprotein; HOMA IR: homeostasis model assessment of insulin resistance; IDF: International Diabetes Federation; LDL: low-density lipoprotein; MetS: metabolic syndrome; NCEP: National Cholesterol Education Programme; NSAIDs: non-steroidal anti-inflammatory drugs; OR: odds ratio; QUICKI: quantitative insulin sensitivity check index; RA: rheumatoid arthritis; TC: total cholesterol; TG: triglycerides; TNF: tumour necrosis factor; WHO: World Health Organization.

## Competing interests

The authors declare that they have no competing interests.

## Authors' contributions

TET analysed and interpreted the data and drafted the manuscript. VFP acquired, analysed and interpreted the data. HJ drafted the manuscript. KMD acquired the data. GDK made substantial contributions to the conception and design of the study and revised the draft manuscript. All authors read and approved the final manuscript.
